# Colonizing Bacteria Aggravate Inflammation, Cytotoxicity and Immune Defense During Influenza A Virus Infection

**DOI:** 10.3390/ijms26115364

**Published:** 2025-06-03

**Authors:** Liane Giebeler, Christina Ehrhardt, Antje Häder, Thurid Lauf, Stefanie Deinhardt-Emmer, Bettina Löffler

**Affiliations:** 1 Section of Experimental Virology, Institute of Medical Microbiology, Center for Molecular Biomedicine (CMB), Jena University Hospital, 07745 Jena, Germany; lgiebeler@e.mail.de (L.G.); christina.ehrhardt@med.uni-jena.de (C.E.); 2 Institute of Medical Microbiology, Jena University Hospital, 07747 Jena, Germany; antje.haeder@med.uni-jena.de (A.H.); thurid.lauf@med.uni-jena.de (T.L.); stefanie.deinhardt-emmer@med.uni-jena.de (S.D.-E.); 3 Cluster of Excellence Balance of the Microverse, Friedrich Schiller University Jena, 07743 Jena, Germany

**Keywords:** commensal bacteria, facultative pathogenic bacteria, influenza A virus, respiratory microbiome, cytotoxicity, cell culture model, macrophages

## Abstract

A diverse bacterial community colonizes the respiratory system, including commensals such as *Staphylococcus epidermidis (S. epidermidis)* and *Streptococcus salivarius (S. salivarius)*, as well as facultative pathogens like *Staphylococcus aureus (S. aureus)*. This study aimed to establish a colonized cell culture model to investigate the impact of these bacteria on influenza A virus (IAV) infection. Respiratory epithelial cells were exposed to *S. epidermidis*, *S. salivarius*, or *S. aureus*, using either live or heat-inactivated bacteria, followed by IAV infection. Cell integrity was assessed microscopically, cytotoxicity was measured via LDH assay, and inflammatory responses were analyzed through cytokine expression. Additionally, macrophage function was examined in response to bacterial colonization and IAV infection. While commensals maintained epithelial integrity for 48 h, *S. aureus* induced severe cell damage and death. The most pronounced epithelial destruction was caused by coinfection with *S. aureus* and IAV. Notably, commensals did not confer protection against IAV but instead enhanced epithelial inflammation. These effects were dependent on live bacteria, as inactivated bacteria had no impact. However, prior exposure to *S. epidermidis* and *S. salivarius* improved macrophage-mediated immune responses against IAV. These findings suggest that while individual commensals do not directly protect epithelial cells, they may contribute to immune training and enhance lung defense mechanisms.

## 1. Introduction

The nasopharyngeal area is colonized by a multitude of bacteria that contribute to the microbiome on our epithelial surfaces. The respiratory microbiome is not only thought to contribute to the overall health of the lung, but also to the defense against respiratory infections [1].

*Staphylococcus epidermidis* (*S. epidermidis*) and *Streptococcus salivarius* (*S. salivarius*) are typical commensals of the nasopharyngeal area and are supposed to be of low virulence [2,3]. Involvement of these bacteria in infections is rare, though endocarditis and foreign material-related infections have been reported [4]. *S. epidermidis* has been detected in great abundance in the nasal mucosa [5]. The bacterium was found to inhibit *S. aureus* and *Moraxella catarrhalis* growth as well as influenza virus replication in vitro, yet knowledge on the relevance of these bacterial interactions in vivo is largely lacking [5,6]. Another member of the respiratory microbiome is *S. salivarius*, which can already be found in the nasopharynx of infants [7]. It largely lacks Streptococcus-typical virulence factors including hemolytic properties and is therefore considered to be of low virulence [8]. Some probiotic properties of *S. salivarius* have been described, e.g., its ability to reduce the amount of recurrent otitis media [9,10].

*Staphylococcus aureus* (*S. aureus*) is a facultative pathogenic or opportunistic bacterium and is known to colonize the nasopharyngeal area and the skin of many individuals. About a third of the healthy population is colonized by *S. aureus*, mostly without developing a disease [11,12]. This colonization can, nevertheless, be the source for severe infections such as pneumonia, endovascular, or soft tissue infections, when the bacterium enters the bloodstream or host tissue structures [13,14]. *S. aureus* possesses a multitude of virulence factors, which enable the bacteria to adhere to host structures, to spread within tissues, to escape of the immune system, and to establish persisting infections [15]. Treatment of *S. aureus * infections can be extremely difficult in severe and persisting infections and is complicated by antibiotic resistance [16]. Furthermore, an effective vaccine is not available [15].

Aside from bacteria, viruses are another major cause for infections, and the epithelial surface (including its microbiome) represents the first line of defense against viral pathogens [17]. A frequent and serious respiratory pathogen is the influenza A virus (IAV), which causes high morbidity and mortality rates in the winter seasons [18]. Young children, elderly individuals, and immunocompromised persons are at high risk of developing severe infection courses [19]. While a vaccination against influenza is available and recommended, it does not provide full protection and depends on a functioning immune system to be effective [20]. In particular, alveolar macrophages and expressed cytokines play a role in the defense against an IAV infection. Macrophages are the primary sentinel cells within the lung, as they play an important role in limiting viral replication and modulating local and adaptive immune responses [21]. Therefore, additional preventive strategies to protect high-risk patients and strengthen their immune functions are warranted. Some components of the respiratory microbiome have been shown to possess possible protective features against influenza viruses. For instance, matrix-binding proteins from *S. epidermidis* have been found to reduce the infectivity of various influenza viruses [22]. However, detailed knowledge of microbial interactions and clinical concepts/recommendations to manipulate the respiratory microbiome for protection against respiratory infections is largely lacking.

The present study was designed to investigate the impact of colonizing commensal bacteria compared to facultative pathogenic bacteria in a respiratory IAV infection model. For this purpose, we established a cell culture system colonized with defined bacteria. We evaluated the direct effect of the colonizing bacteria on the epithelial cell integrity, inflammation, and their impact on an IAV infection. Furthermore, we tested the effect of commensal bacteria on macrophages during an IAV infection.

## 2. Results

### 2.1. S. aureus Harms the Integrity of the Epithelial Cell Layer Compared to the Commensal Bacteria S. epidermidis and S. salivarius

**Figure 1:**
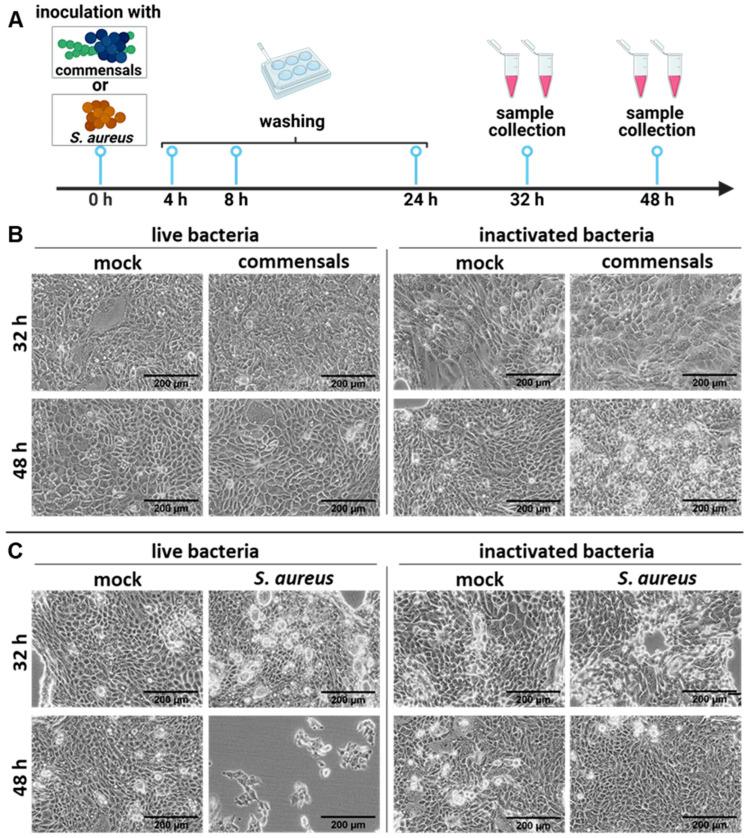
*S. aureus* harms the integrity of the epithelial cell layer compared to *S. epidermidis* and *S. salivarius*. (**A**) Study design of the colonization of Calu-3 cells with live or inactivated commensal (*S. salivarius* and *S. epidermidis*) and opportunistic bacteria (*S. aureus)*. Light microscopy pictures of Calu-3 cells inoculated with either (**B**) *S. salivarius* and *S. epidermidis* (0.00001 MOI for live commensals; 1.85 × 10^6^ bacteria/well for inactivated *S. salivarius* and 2.1 × 10^6^ bacteria/well for inactivated *S. epidermidis*) or (**C**) *S. aureus* (0.00002 MOI for live *S. aureus*; 2 × 10^8^ bacteria/well for inactivated *S. aureus*). The cells were washed twice with PBS at 4 and 8 h post-inoculation (p. io.), respectively, to reduce the amount of non-internalized bacteria. Microscopic analysis was performed 32 or 48 h p. io. as indicated. Scale bars represent 200 µm.

To elucidate the impact of bacterial members of the respiratory tract microbiome, we aimed to establish a minimalist colonized cell culture model. For this purpose, the human lung epithelial cell line Calu-3 was co-cultured with the commensal bacteria *S. salivarius* and *S. epidermidis* for up to 48 h. In detail, Calu-3 cells were seeded 48 h before the experiment. Cells were inoculated with either live (MOI of 0.00001) or inactivated *S. salivarius* and *S. epidermidis.* The amount of inactivated bacteria used was the maximum number of bacteria measured during experiments with live bacteria. At 4, 8, and 24 h post-inoculation (p. io.), the cells were washed two times with PBS to reduce the bacterial load. At 32 and 48 h p. io., samples and microscopy pictures were taken (Figure 1A).

As shown by the light microscopy, no destruction of the cell layer was visible upon co-cultivation with live commensal bacteria, neither 32 nor 48 h p. io. (Figure 1B, left panel). Additionally, co-cultivation with inactivated bacteria revealed similar results (Figure 1B, right panel). These findings demonstrate the low virulence of the commensal bacteria and show the establishment of a stable and intact cell culture system containing live commensal bacteria for up to 48 h.

To further test the impact of opportunistic bacteria, the model system was colonized with *S. aureus*. In contrast to the previous results, we observed cell layer disturbances in the samples treated with live *S. aureus*, which developed into a largely destroyed epithelial cell monolayer 48 h p. io. (Figure 1C, left panel). The addition of inactivated *S. aureus* did not affect the epithelial cell monolayer (Figure 1C, right panel). Therefore, metabolically active bacteria and virulence factor expression are required to fully destroy the epithelial cell layer, while cell wall components or bacterial remnants are not sufficient.

### 2.2. Colonization of the Epithelial Layer with Commensal Bacteria Does Not Protect from an Influenza Virus Infection, but Causes Inflammation and Enhances Viral Titers

**Figure 2:**
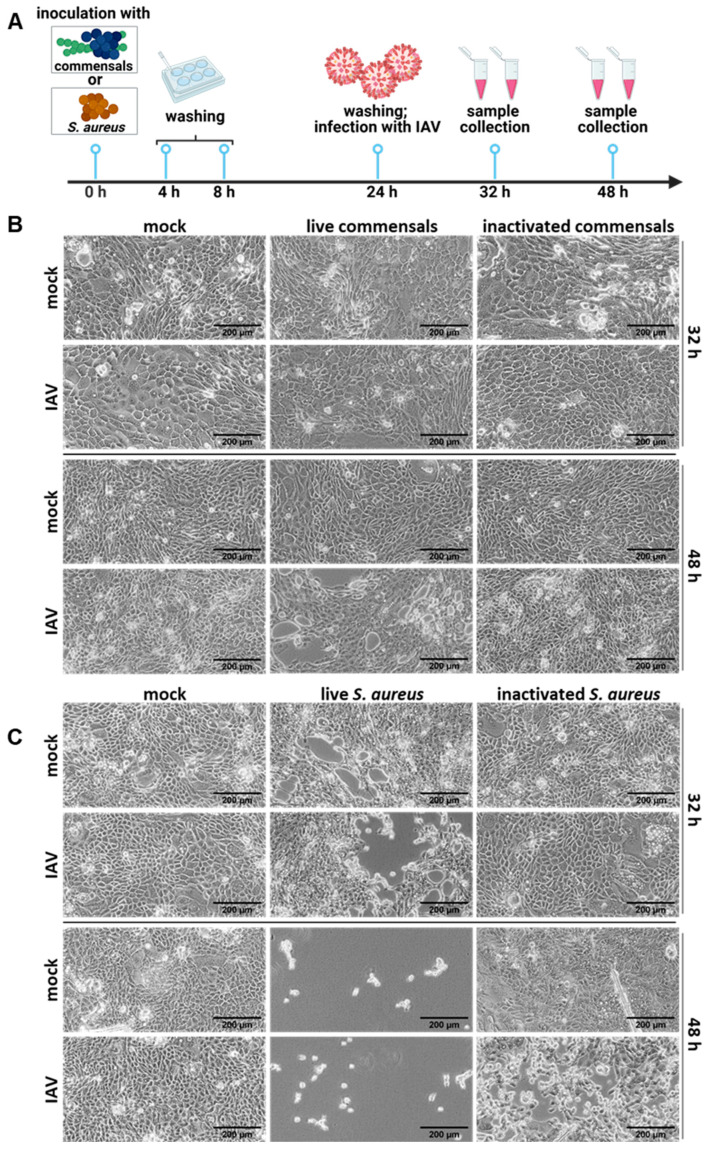
Colonization of the epithelial layer with live bacteria does not protect from an influenza virus infection, but adds to the epithelial damage. (**A**) Study design of the colonization of Calu-3 cells with live or inactivated commensal and opportunistic bacteria, followed by an influenza A virus infection (IAV). (**B**) Light microscopy pictures of Calu-3 cells inoculated with either *S. salivarius* and *S. epidermidis* (0.00001 MOI for live commensals; 1.85 × 10^6^ bacteria/well for inactivated *S. salivarius* and 2.1 × 10^6^ bacteria/well for inactivated *S. epidermidis*) or (**C**) *S. aureus* (0.00002 MOI for live *S. aureus*; 2 × 10^8^ bacteria/well for inactivated *S. aureus*). The cells were washed twice with PBS at 4 and 8 h p. io., respectively, to reduce the amount of non-internalized bacteria. Infection with IAV was performed 24 h p. io. Microscopic analysis was performed 32 h or 48 h p. io. Scale bars represent 200 µm.

To investigate the impact of colonizing commensals on an influenza virus infection, we challenged the cell culture system with an IAV infection, infecting the system with the Influenza virus A/Puerto Rico/8/34 (H1N1). For this, the same procedure as described above was performed, but at 24 h p. io., IAV was added at an MOI of 5 for the 32 h samples and at an MOI 1 for the 48 h samples. This allowed us to examine the effects after one (32 h co-cultivation with 8 h IAV infection) and several (48 h co-cultivation with 24 h IAV infection) IAV replication cycles (Figure 2A).

As evidenced by the microscopic analysis, single IAV infection did not cause visible changes in the cell morphology due to the relatively short infection time (Figure 2). Yet, colonization with commensal bacteria preceding the IAV infection caused some damage in the monolayer at 48 h p. io. (24 h viral infection), while no obvious changes were visible at 32 h p. io. (8 h viral infection) (Figure 2B). These alterations were not observed in samples treated with inactivated bacteria (Figure 2B). Additionally, quantification of the bacterial titer (colony-forming units (CFUs)) revealed high bacterial loads intra- and extracellularly for both bacteria. The infection with IAV did not affect the bacterial titers (Supplementary Figure S1).

The destructive effect on the cell monolayer was much more pronounced when *S. aureus* was used as colonizing bacterium. Here, severe damage of the cell layer could already be observed at 32 h p. io. (8 h viral infection), and by 48 h p. io., the cell layer was completely destroyed (Figure 2C. Samples treated with inactivated bacteria remained morphologically intact (Figure 2C).

From our results, we can conclude that the epithelial cell layer is severely harmed by *S. aureus*, but the commensal bacteria *S. epidermidis* and *S. salivarius* also contribute to cell damage during an influenza virus infection, albeit to a much lesser extent. The addition of inactivated bacteria did not affect the cell monolayers, indicating that metabolically active bacteria are required to induce cell damage.

**Figure 3:**
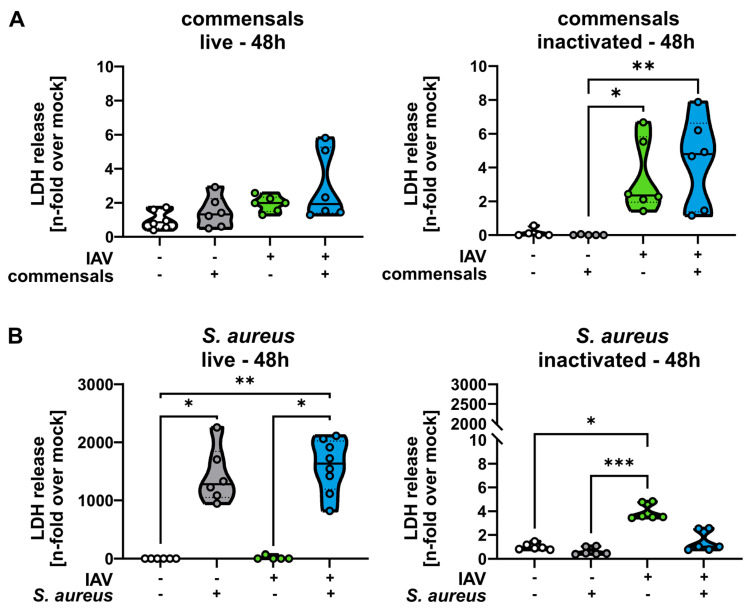
Live *S. aureus* (but not commensal bacteria) increases the LDH release of epithelial cells. Calu-3 cells were inoculated with either (**A**) *S. salivarius* and *S. epidermidis* or (**B**) *S. aureus* as live or inactivated bacteria as indicated, washed twice with PBS at 4 and 8 h, and infected with IAV (1 MOI) at 24 h p. io. Samples were taken at 48 h p. io. Cell death rate was assessed with LDH assays 48 h p.io. Data shown represent the means ± SD of at least three independent experiments with two technical replicates. Statistical analysis was performed with Kruskal–Wallis test and Dunn’s multiple comparison test. * *p* < 0.05, ** *p* < 0.01, *** *p* < 0.001.

To confirm and quantify the microscopic analysis shown in Figure 1 and Figure 2, we measured the LDH release as an indicator for cytotoxicity in the cells at 48 h p. io. As expected, samples treated with live or inactivated commensals showed no significant increase in LDH release. Only in IAV-infected samples could slightly increased LDH levels be measured (Figure 3A). Samples treated with live *S. aureus*, however, showed a much stronger effect, as a highly significant LDH release in comparison to mock- or IAV-infected samples could be measured (Figure 3B). Again, the effect could not be reproduced by inactivated *S. aureus*.

**Figure 4:**
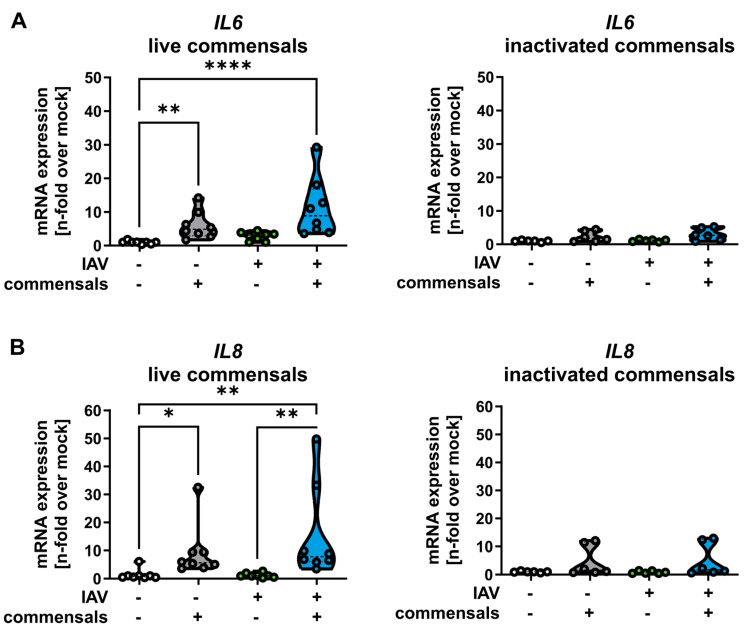
Live commensal bacteria induce the expression of interleukin-6 (IL-6) and -8 (IL-8) in epithelial cells. Calu-3 cells were inoculated with either *S. salivarius* and *S. epidermidis* or *S. aureus* as live or inactivated bacteria, as indicated, washed twice with PBS at 4 and 8 h, and infected with IAV (1 MOI) at 24 h p. io. RNA was extracted at 32 h p. io. with either live (left panel) or inactivated (right panel) commensal bacteria, followed by cDNA synthesis and qRT-PCR for IL-6 (**A**) and IL-8 (**B**). Data represent the median of at least three independent experiments with two technical replicates. Statistical significance was determined by Kruskal–Wallis test and Dunn’s multiple comparison test. * *p* < 0.05; ** *p* < 0.01; **** *p* < 0.0001.

As commensal bacteria hardly affected cell viability, we next measured the pro-inflammatory activation of epithelial cells caused by the stable bacterial colonization. For this, cytokine profiles in the presence and absence of the colonizing bacteria and IAV infection were examined (Figure 4). Our data indicate that colonizing commensal bacteria stimulated the expression of pro-inflammatory cytokines interleukin-6 (IL-6, Figure 4A) and interleukin-8 (IL-8, Figure 4B). 

**Figure 5:**
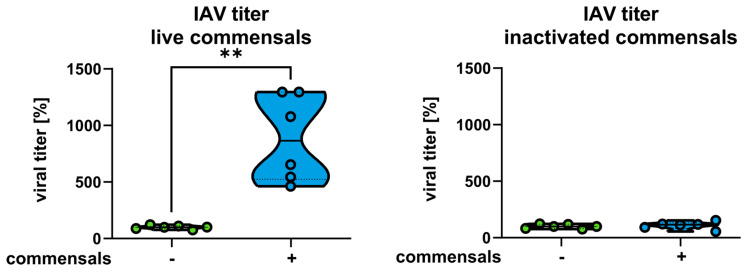
Commensal bacteria enhance the viral titers in the supernatants of epithelial cells. Calu-3 cells were inoculated with control medium compared to commensals (*S. salivarius* and *S. epidermidis*) as live or inactivated bacteria, as indicated, washed twice with PBS at 4 and 8 h, and infected with IAV (1 MOI) at 24 h p. io. IAV titers were determined by standard plaque assays at 32 h p. io. for live (left panel) or inactivated (right panel) commensals. Titers are shown in percent with the mean of the single infection set as 100%. Data represent means + SD of at least three independent experiments with two technical replicates. Statistical significance was determined by Mann–Whitney test ** *p* < 0.01.

Finally, we aimed to investigate whether the inflammatory response affects the clearance of viral particles in the epithelial cells. For this purpose, we determined the extracellular viral load by standard plaque assays. The results show significantly increased viral titers at 32 h p. io. in host cells colonized with live commensal bacteria compared to cells without colonization (Figure 5). Interestingly, the incubation with inactivated bacteria followed by an IAV infection did not change the virus titer (Figure 5).

In summary, we found that live commensal bacteria do not damage epithelial cells, but during an IAV infection, they enhance cell damage as well as the inflammatory response and viral titers.

### 2.3. Inactivated Colonizing Commensal Bacteria Act as Immune Training for Human Monocyte-Derived Macrophages

**Figure 6:**
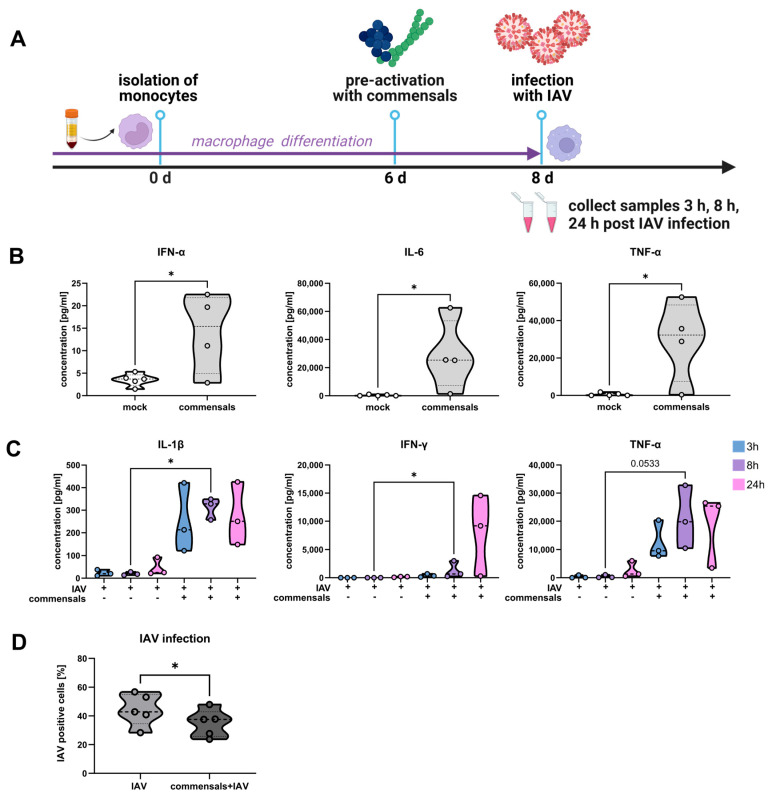
Commensal bacteria pre-activate macrophages and enhance the clearance of IAV infection. (**A**) Study design of monocyte-derived macrophages that were pre-stimulated with the inactivated commensal bacteria *S. epidermidis* (MOI 0.5) and *S. salivarius* (MOI 0.5) for 2 d prior to the infection with IAV (MOI 1). (**B**) The antiviral cytokine response was measured in the supernatants of monocyte-derived macrophages with or without pre-stimulation with inactivated commensals for 2 d and (**C**) with subsequent IAV infection for 3 h, 8 h, and 24 h via multiplexed immunoassay (*n* = 3; Kruskal–Wallis test and Dunn’s multiple comparison test). (**D**) IAV-positive macrophages were quantified 24 h post IAV infection by flow cytometry with or without pre-stimulation with inactivated commensal bacteria (*n* = 5, paired *t*-test). * *p* < 0.05.

To investigate the impact of colonizing commensal bacteria on immune cells, we incubated primarily isolated monocytes during the differentiation process into macrophages (hMdM) with inactivated bacteria of *S. epidermidis* and *S. salivarius* (Supplementary Figure S2 and Figure 6A). This resulted in pre-activation of the hMdM after 2 d, characterized by enhanced secretion of the pro-inflammatory cytokines IFN-α, IL-6, and TNF-α (Figure 6B).

In the published literature, it has been proposed that the lung microbiome could act as immune stimulation and training for the immune system [23,24,25]. In our experiments, we challenged the macrophages after the pre-stimulation with commensal bacteria with an IAV infection. We measured the cytokine response and found that IL-1β, TNF-α, and IFN-γ were significantly higher in macrophages pre-activated with the commensal bacteria than in macrophages without pre-activation at 3 h, 8 h, and 24 h post IAV infection, respectively (Figure 6C).

Furthermore, our results clearly indicate that less virus particles were able to replicate in pre-activated macrophages, as we observed significantly less IAV-positive macrophages upon flow cytometry assessment (Figure 6D).

In summary, we found that the commensal bacteria *S. epidermidis* and *S. salivarius* can function as an immune stimulus for macrophages that enhance the inflammatory response and viral clearance during an IAV infection.

## 3. Discussion

The epithelial surfaces of the respiratory tract are colonized by a diverse flora, which has been attributed to have protective functions against respiratory infections, including viral and bacterial pathogens [22,23,26]. Some of these protective effects are explained by direct inhibitory activity of the commensal bacteria against pathogens, such as the production of inhibitory components [22,24]. Other protective mechanisms include an enhanced lung immunity induced by commensal bacteria [23].

Our study provides evidence that, on the one hand, colonizing commensal bacteria outside their usual microbiome are able to harm epithelial cells, which results in an aggravated infection scenario during an influenza virus infection. On the other hand, commensals can prime macrophages, which contributes to clear an IAV infection.

We established a cell culture system colonized with the typical commensal bacteria *S. epidermidis* and *S. salivarius*. The system remained intact without significant cell death induction for up to 48 h. In contrast, the facultative pathogenic bacterium *S. aureus* harms the epithelial layer upon colonization. This effect was dependent on viable bacteria and is most likely attributed to the multitude of virulence factors expressed by *S. aureus*, as well as the intracellular lifestyle that contributes to cytotoxicity [25,27].

Many facultative pathogenic bacteria, including *S. aureus*, are recognized as a common part of the microbiome in the nasopharyngeal area [11,28]. Interestingly, these colonizing *S. aureus* strains retain their full virulent capacity and ability to cause tissue damage upon infection. In previously published work, we demonstrated a similar level of cytotoxicity and invasive ability in both colonizing and pathogenic *S. aureus* strains [29,30]. Accordingly, the restriction and control of facultative pathogens by a surrounding microbiome are essential and have been described as important factors of the host immune system to control infections [17]. Recent work suggests that the composition of the nasal microbiota plays a major role in promoting or inhibiting *S. aureus* colonization and infection [28]. Some members of the microbiome with possible control functions have been identified; examples include *Staphylococcus lugdunensis* in the nasal area or the probiotic *Bacillus subtilis* within the gut [31,32].

To study the interaction between the colonizing bacteria and a pathogen, we further challenged our colonized epithelial cell culture system with an influenza virus infection. *S. aureus* induced the strongest cytotoxic and destructive effects compared to *S. epidermidis* and *S. salivarius*. Nevertheless, *S. epidermidis* and *S. salivarius* also affected the epithelial layer during an influenza virus infection, mainly by inducing inflammation. These findings suggest that commensal bacteria with low virulence are not entirely non-pathogenic, but display characteristics of infectious bacteria, e.g., due to their cell wall components or some secreted factors [33]. The virulence of commensal bacteria is apparently also controlled by microbial interactions as well as the immune system. Consequently, our experiments demonstrate that the described protective effects of the microbiome against an influenza virus infection cannot be reproduced by defined single bacteria in a single cell system [34].

Aside from the bacterial effects on epithelial cells, we could confirm that colonizing commensal bacteria function as immune training for macrophages. The recent literature shows that bacteria exposed to immune cells, in particular macrophages, could function as immune training and enhance the phagocytic capacity. This phenomenon was described with probiotic-based nanoparticles and commensal bacteria to increase the macrophage’s phagocytic ability and protect against viral infection in mice [35,36].

Macrophages, as key sentinels of the immune system in the lung, exhibit a remarkable ability to detect and respond to microbial stimuli. The observation of a pre-activation of these macrophages after exposure to non-viable bacteria indicates a form of immune memory or preparedness, priming them for a heightened response upon encountering subsequent pathogens [37]. This phenomenon of pre-activation suggests a potential mechanism to enhance the immune defense against incoming pathogens. The primed state of these macrophages could enable a more rapid and robust response upon encountering invading pathogens, such as influenza viruses, thereby enhancing the host’s ability to combat infections.

Taken together, we established an epithelial cell culture model that can be colonized by commensal bacteria and remained stable for 48 h. It is a clear limitation of our model system that it is restricted to defined commensals and does not represent the full complex microbiome. Yet, this model can be applied to test the degree of virulence of colonizing bacteria and the interaction of colonizing bacteria with epithelial cells. Nevertheless, the commensals showed no protective effect during an influenza virus infection scenario, and the implementation of *S. aureus* caused significant rates of cell destruction. Our findings show that the protective properties of the microbiome cannot be reproduced through specific interactions of individual colonizing bacteria in a cell culture system without immune cells. To take advantage of the microbiome as a therapeutic tool, a detailed understanding of the different microbes, the complex microbial interactions, and their interaction with professional immune cells is required.

## 4. Materials and Methods

### 4.1. Cells

Calu-3 cells (provided by the laboratory of Stephan Ludwig, Münster, Germany) were cultivated in Dulbecco’s modified Eagle medium (DMEM, high-glucose Sigma-Aldrich, Taufkirchen, Germany) supplemented with 10% fetal calf serum (FCS, Anprotec, Bruckberg, Germany) at 37 °C, 5% CO_2_, and 95% humidity.

### 4.2. Virus Propagation

Influenza virus A/Puerto Rico/8/34 (H1N1) (provided from the viral stock collection of Stephan Ludwig, Münster, Germany) was used for all experiments. The virus was propagated on Madin–Darby canine kidney II (MDCK II) cells [38].

### 4.3. Bacterial Culture

One bacterial colony of either *S. salivarius subsp. salivarius strain* C699 [S30D], *S. epidermidis*, or methicillin-resistant *S. aureus* strain USA 300 was transferred into either 5 (for experiments with live bacteria) or 25 mL (for experiments with inactivated bacteria) brain heart infusion (BHI, Oxoid, Wesel, Germany) and incubated overnight at 37 °C. The titer of the cultures was assessed via OD600 measurement using a BioPhotometer (Eppendorf, Hamburg, Germany; OD600 1 of *S. salivarius* = 3.33 × 10^7^ CFU/mL; OD600 1 of *S. epidermidis* = 1.25 × 10^8^ CFU/mL; OD600 1 of *S. aureus* = 5 × 10^8^ CFU/mL).

### 4.4. Bacterial Inactivation

The 25 mL overnight cultures of *S. salivarius*, *S. epidermidis*, and *S. aureus* were centrifuged with 4612× *g* for 10 min at room temperature (RT). After removal of the supernatant, 10 mL of phosphate-buffered saline (PBS; Roth, Karlsruhe, Germany) containing 4% formaldehyde (diluted from a 37.5% solution; Roth, Karlsruhe, Germany) was added and the resulting suspension was incubated at RT for 2 h. After centrifugation with the same conditions as above, the supernatant was removed and PBS was added, followed by centrifugation with 2200× *g* for 10 min at RT after re-suspension. After three repetitions of this step, the inactivated bacteria were finally suspended in 1.5 mL PBS, and the titer was determined by OD600 measurement.

The successful inactivation of the bacteria was ensured by plating 100 µL of the bacteria on Müller–Hinton (MH) agar plates followed by overnight incubation at 37 °C.

### 4.5. Cell Culture and Viral Infection

At 48 h prior to the experiment, Calu-3 cells were seeded in 6-well plates in DMEM supplemented with 10% FCS and incubated at 37 °C, 5% CO_2_, and 95% humidity. The cell density was 8 × 10^5^ cells per well.

At 0 h, the medium was removed from the cells, followed by two washing steps and the addition of 1.5 mL RPMI supplemented with 10% FCS containing bacteria sufficient for the multiplicity of infection (MOI) indicated in the results. This was followed by 4 h of incubation with the same conditions as above. Subsequently, the cells were washed 2× with PBS, and 1.5 mL of RPMI supplemented with 10% FCS was added. This procedure was repeated at 8 h post-inoculation (p.io).

At 24 h p.io., 500 µL of infection medium (RPMI supplemented with 0.6% bovine serum albumin (BSA; Roth, Karlsruhe, Germany), 1 mM of MgCl_2_, 0.9 mM of CaCl_2_, and 167 ng/mL of trypsin TPCK (Sigma-Aldrich, Taufkirchen, Germany)) containing the appropriate amount of IAV for the MOIs indicated in the results were added. The medium was removed after incubation at 37 °C, 5% CO_2_, and 95% humidity for 30 min and 1.5 mL of new infection medium was added, followed by incubation for either 8 or 24 h in the same conditions as described above. Subsequently, cells and supernatants were collected and used in the further analyses.

Mock control means that the cells have been treated with identical media for the identical times as the infected and co-cultivated samples, but without the addition of any bacteria or virus.

### 4.6. Light Microscopy

After removal of the medium, the cells were washed one time with PBS, followed by the addition of fresh PBS and the acquisition of light microscopy pictures 32 and 48 h p. io. All images were taken by an Axio-Vert. A1 microscope (ZEISS, Jena, Germany) and processed with ZEN core 3.0 as well as ImageJ 1.53a.

### 4.7. Determination of Extracellular Bacterial Titers

A volume of 100 µL of the cell culture supernatants, collected 32 h p. io. from the experiment described in section “cell culture and viral infection”, was plated on Müller–Hinton (MH, Roth, Karlsruhe, Germany) agar plates. Depending on the sample, the supernatant was either diluted in PBS or left undiluted. The plates were then incubated for approximately 48 h with *S. salivarius* and *S. epidermidis*, after which the colony formation was quantified to determine the extracellular titer.

### 4.8. Determination of Intracellular Bacterial Titers

Cells, treated as described in the section “cell culture and viral infection”, were incubated in RPMI medium supplemented with 10% fetal calf serum (FCS, Merck) and 2 µg/mL of Lysostaphin (WAK-Chemie, Steinbach, Germany) for 20 min at 32 h p. io. Following incubation, the cells were washed twice with phosphate-buffered saline (PBS; Roth, Karlsruhe, Germany). Subsequently, cold water was added, and the cells were incubated at 4 °C for 10 min. The cell lysates were collected, centrifuged at 7000 rpm for 10 min at RT, and pellets were resuspended in 1 mL PBS. The lysates were then plated on MH (Roth, Karlsruhe, Germany) agar plates, either diluted in PBS or left undiluted, depending on the sample. The plates were incubated for approximately 48 h with *S. salivarius* and *S. epidermidis*, after which the colony formation was assessed to determine the extracellular titer.

### 4.9. RNA Extraction

The cells were detached with trypsin-EDTA solution (Anprotec, Bruckberg, Germany) and pelleted using centrifugation at 2200× *g* for 10 min. The RNA was extracted using the RNeasy Mini Kit (Qiagen, Hilden, Germany). The resulting RNA concentration and purity were assessed via OD_260_ measurement.

### 4.10. cDNA Synthesis

cDNA synthesis was performed with 400 ng of RNA per sample using the QuantiTectTM kit (Qiagen, Hilden, Germany) in accordance with the supplier’s instructions.

### 4.11. qRT-PCR

For the qRT-PCRs, the QuantiNovaTM SYBR^®^ green PCR kit (Qiagen, Hilden, Germany) was used. First, 3 µL of 1:3 diluted cDNA was mixed with 7 µL master mix (composed of 0.5 µL of RNase-free double-distilled water (ddH_2_O), 0.75 µL of forward (fwd) primer (10 mM), 0.75 µL of reverse primer (rev) (10 mM), and 5 µL of SYBR green per sample). The qRT-PCR was performed using a 72-well RotorGene (Qiagen, Hilden, Germany) with the following cycle conditions: after an initial heating to 95 °C for 2 min, 40 cycles with 95 °C for 5 s and subsequent 60 °C for 10 s followed. Termination of the qRT-PCR cycle was achieved by a gradual temperature increase in 1 °C increments every 5 s, starting at 60 °C and ending at 95 °C. The sequences of the primers used were the following: GAPDH_fwd 5′-CTCTGCTCCTCCTGTTCGAC-3′, GAPDH_rev 5′-CAATACGACCAAATCCGTTGAC-3′; IL-6_fwd 5′-CAGCCCTGAGAAAGGAGACATG-3′, IL-6_rev 5′-GCATCCATCTTTTTCAGCCATC-3′; IL-8_fwd 5′-ATGACTTCCAAGCTGGCCGTGGCT-3′, IL-8_rev 5′-TCTCAGCCCTCTTCAAAAACTTCT-3′.

### 4.12. Lactate Dehydrogenase (LDH)-Assay

LDH analysis was performed with the CyQUANT^TM^ LDH Cytotoxicity Assay (Invitrogen, Dreieich, Germany) in accordance with the supplier’s instructions. For the absorption measurement at 490 and 680 nm, a FLUOstar^®^ Omega (BMG LABTECH, Ortenberg, Germany) was used, and the data were analyzed with Omega 5.50 R4 and MARS 3.32 R5.

### 4.13. Monocyte Isolation, Differentiation, and Infection

Buffy coats were commercially obtained from healthy donors under the age of 25, provided by the Institute of Transfusion Medicine, University Hospital Jena. Human peripheral blood mononuclear cells (PBMCs) were isolated using a Histopaque^®^-1077 (Sigma Aldrich, St. Louis, MO, USA) density gradient and cultured in RPMI 1640 (Thermo Fisher Scientific, Waltham, MA, USA) for 2 h at 37 °C. After washing with phosphate-buffered saline (PBS, Thermo Fisher Scientific), the attached monocytes were differentiated into macrophages using RPMI 1640 supplemented with 10% HSA (Pan Biotech, Aidenbach, Germany), 1% penicillin/streptomycin (P/S, Lonza, Basel, Switzerland), and 0.01% recombinant GM-CSF (PeproTech, Hamburg, Germany) with a change in media every 2 days. On day 6, macrophages were stimulated with commensals, containing formaldehyde-inactivated *S. salivarius* and *S. epidermidis* at an MOI of 0.5 for each bacterial strain (see above for the bacterial inactivation protocol). After 48 h, IAV was added to the media at an MOI of 1 and incubated for 3 h, 8 h, and 24 h.

### 4.14. Protein Analysis

The quantification of cytokines in the supernatants of the infected macrophages was performed via multiplexed immunoassay using the LEGENDplex Human Inflammation Panel I Kit (BioLegend, San Diego, CA, USA) according to the manufacturer’s protocol. The samples were analyzed using BD FACSSymphony A1 Flow Cytometry (BD Bioscience, Heidelberg, Germany). Cytokine concentrations were calculated with the LEGENDplex Data Analysis Software Suite (v.2024-06-15).

### 4.15. Flow Cytometry

To prove the differentiation of monocytes into hMDM, cells were incubated with Accutase (Sigma-Aldrich, Merck, Darmstadt, Germany) for 20 min. Cells were further detached using a cell scraper and washed with PBS. To discriminate dead cells, the suspension was incubated with BD Fixable Viability Stain 780 (BD Biosciences, Franklin Lakes, NJ, USA) at a 1:1000 dilution in PBS at room temperature in the dark for 15 min. Cells were washed and resuspended in BD Staining Buffer (BD Biosciences) containing BV786 Mouse Anti-Human CD80 and APC Mouse Anti-Human CD86 (both BD Biosciences, diluted 1:100), and incubated for 20 min at 4 °C. After washing with PBS, the cells were fixed and permeabilized with BD Fixation/Permeabilization Solution (BD Biosciences) for 20 min at 4 °C. The fixed cells were washed with BD Perm/Wash Buffer (BD Biosciences) and stained with PE Mouse Anti-Human CD68 Set antibody (BD Biosciences) diluted 1:100 in BD Perm/Wash Buffer and incubated for 30 min at 4 °C. Following a final wash, cells were resuspended in Perm/Wash Buffer and mean fluorescence intensities were quantified. Differentiated hMdM are characterized by increased expression of CD80, CD86, and CD68 (Supplementary Figure S2a).

To quantify IAV-positive hMdM, the cells were detached, stained for live/death cells and fixed/permeabilized as described above and then incubated with the Influenza A Nucleoprotein Antibody (abcam, Cambridge, UK) for 30 min at 4 °C. After washing, cells were resuspended and quantified. All measurements were performed using a BD FACSymphony A1 Flow Cytometer. Data analysis was performed using FlowJo v10.8.

### 4.16. Immunofluorescence

For immunostaining of hMdM (Supplementary Figure S2b), monocytes were seeded on coverslips and differentiated as described above. The hMdMs were fixed with 4% PFA for 1 h at 37 °C, then washed and incubated in blocking buffer (3% BSA in PBS) for 1 h at room temperature. Actin was stained with Alexa Fluor 488 Phalloidin (Invitrogen, diluted 1:400) for 1 h at room temperature in blocking buffer. Coverslips were then mounted using DAPI Fluoromount-G (Southern Biotech, Birmingham, AL, USA), and imaging was conducted using an AxioObserver Z.1 + Apotome 2 microscope (Carl Zeiss, Oberkochen, Germany).

### 4.17. Statistics and Data Visualization

All graphs and statistical analyses were conducted with PRISM 9.3.1. Statistical analyses was performed as described in the figure legends. Illustrations were created in https://www.biorender.com/ (accessed on 6 February 2025).

## Data Availability

The data presented in this study are available in the present article.

## Supplementary Materials

The following supporting information can be downloaded at: https://www.mdpi.com/article/10.3390/ijms26115364/s1.

## References

[1] Wypych T.P., Wickramasinghe L.C., Marsland B.J. (2019). The influence of the microbiome on respiratory health. Nat. Immunol..

[2] Otto M. (2009). Staphylococcus epidermidis—the ‘accidental’ pathogen. Nat. Rev. Microbiol..

[3] Delorme C., Abraham A.L., Renault P., Guédon E. (2015). Genomics of Streptococcus salivarius, a major human commensal. Infect. Genet. Evol..

[4] Oberbach A., Schlichting N., Hagl C., Lehmann S., Kullnick Y., Friedrich M., Köhl U., Horn F., Kumbhari V., Löffler B. (2023). Four decades of experience of prosthetic valve endocarditis reflect a high variety of diverse pathogens. Cardiovasc. Res..

[5] Liu Q., Liu Q., Meng H., Lv H., Liu Y., Liu J., Wang H., He L., Qin J., Wang Y. (2020). Staphylococcus epidermidis Contributes to Healthy Maturation of the Nasal Microbiome by Stimulating Antimicrobial Peptide Production. Cell Host Microbe.

[6] Janek D., Zipperer A., Kulik A., Krismer B., Peschel A. (2016). High Frequency and Diversity of Antimicrobial Activities Produced by Nasal Staphylococcus Strains against Bacterial Competitors. PLoS Pathog..

[7] Carlsson J., Grahnen H., Jonsson G., Wikner S. (1970). Early Establishment of Streptococcus salivarius in the Mouths of Infants. J. Dent. Res..

[8] Burton J.P., Wescombe P.A., Moore C.J., Chilcott C.N., Tagg J.R. (2006). Safety assessment of the oral cavity probiotic Streptococcus salivarius K12. Appl. Environ. Microbiol..

[9] Di Pierro F., Donato G., Fomia F., Adami T., Careddu D., Cassandro C., Albera R. (2012). Preliminary pediatric clinical evaluation of the oral probiotic Streptococcus salivarius K12 in preventing recurrent pharyngitis and/or tonsillitis caused by Streptococcus pyogenes and recurrent acute otitis media. Int. J. Gen. Med..

[10] Passali D., Passali G.C., Vesperini E., Cocca S., Visconti I.C., Ralli M., Bellussi L.M. (2019). The efficacy and tolerability of Streptocococcus salivarius 24SMB and Streptococcus oralis 89a administered as nasal spray in the treatment of recurrent upper respiratory tract infections in children. Eur. Rev. Med. Pharmacol. Sci..

[11] Deinhardt-Emmer S., Sachse S., Geraci J., Fischer C., Kwetkat A., Dawczynski K., Tuchscherr L., Löffler B. (2018). Virulence patterns of *Staphylococcus aureus* strains from nasopharyngeal colonization. J. Hosp. Infect..

[12] Piewngam P., Otto M. (2024). *Staphylococcus aureus* colonisation and strategies for decolonisation. Lancet Microbe.

[13] von Eiff C., Becker K., Machka K., Stammer H., Peters G. (2001). Nasal carriage as a source of *Staphylococcus aureus* bacteremia. N. Engl. J. Med..

[14] Troeman D.P.R., Hazard D., Timbermont L., Malhotra-Kumar S., van Werkhoven C.H., Wolkewitz M., Ruzin A., Goossens H., Bonten M.J.M., Harbarth S. (2023). Postoperative *Staphylococcus aureus* Infections in Patients With and Without Preoperative Colonization. JAMA Netw. Open.

[15] Cheung G.Y.C., Bae J.S., Otto M. (2021). Pathogenicity and virulence of Staphylococcus aureus. Virulence.

[16] Kahl B.C., Becker K., Loffler B. (2016). Clinical Significance and Pathogenesis of Staphylococcal Small Colony Variants in Persistent Infections. Clin. Microbiol. Rev..

[17] Tosta E. (2021). The seven constitutive respiratory defense barriers against SARS-CoV-2 infection. Rev. Soc. Bras. Med. Trop..

[18] Iuliano A.D., Roguski K.M., Chang H.H., Muscatello D.J., Palekar R., Tempia S., Cohen C., Gran J.M., Schanzer D., Cowling B.J. (2018). Estimates of global seasonal influenza-associated respiratory mortality: A modelling study. Lancet.

[19] GBD 2017 Lower Respiratory Infections Collaborators (2020). Quantifying risks and interventions that have affected the burden of lower respiratory infections among children younger than 5 years: An analysis for the Global Burden of Disease Study 2017. Lancet Infect. Dis..

[20] Tanner A.R., Dorey R.B., Brendish N.J., Clark T.W. (2021). Influenza vaccination: Protecting the most vulnerable. Eur. Respir. Rev..

[21] Li H., Wang A., Zhang Y., Wei F. (2023). Diverse roles of lung macrophages in the immune response to influenza A virus. Front. Microbiol..

[22] Chen H.W., Liu P.F., Liu Y.T., Kuo S., Zhang X.Q., Schooley R.T., Rohde H., Gallo R.L., Huang C.M. (2016). Nasal commensal Staphylococcus epidermidis counteracts influenza virus. Sci. Rep..

[23] Brown R.L., Sequeira R.P., Clarke T.B. (2017). The microbiota protects against respiratory infection via GM-CSF signaling. Nat. Commun..

[24] Kikukawa H., Nagao T., Ota M., Takashima S., Kitaguchi K., Yanase E., Maeda S., Hara K.Y. (2023). Production of a selective antibacterial fatty acid against *Staphylococcus aureus* by Bifidobacterium strains. Microbiome Res. Rep..

[25] Fraunholz M., Sinha B. (2012). Intracellular staphylococcus aureus: Live-in and let die. Front. Cell Infect. Microbiol..

[26] Kanmani P., Clua P., Vizoso-Pinto M.G., Rodriguez C., Alvarez S., Melnikov V., Takahashi H., Kitazawa H., Villena J. (2017). Respiratory Commensal Bacteria Corynebacterium pseudodiphtheriticum Improves Resistance of Infant Mice to Respiratory Syncytial Virus and Streptococcus pneumoniae Superinfection. Front. Microbiol..

[27] Otto M. (2014). *Staphylococcus aureus* toxins. Curr. Opin. Microbiol..

[28] Krismer B., Weidenmaier C., Zipperer A., Peschel A. (2017). The commensal lifestyle of *Staphylococcus aureus* and its interactions with the nasal microbiota. Nat. Rev. Microbiol..

[29] Tuchscherr L., Pöllath C., Siegmund A., Deinhardt-Emmer S., Hoerr V., Svensson C.M., Thilo Figge M., Monecke S., Löffler B. (2019). Clinical *S. aureus* Isolates Vary in Their Virulence to Promote Adaptation to the Host. Toxins.

[30] Deinhardt-Emmer S., Haupt K.F., Garcia-Moreno M., Geraci J., Forstner C., Pletz M., Ehrhardt C., Löffler B. (2019). *Staphylococcus aureus* Pneumonia: Preceding Influenza Infection Paves the Way for Low-Virulent Strains. Toxins.

[31] Zipperer A., Konnerth M.C., Laux C., Berscheid A., Janek D., Weidenmaier C., Burian M., Schilling N.A., Slavetinsky C., Marschal M. (2016). Human commensals producing a novel antibiotic impair pathogen colonization. Nature.

[32] Piewngam P., Khongthong S., Roekngam N., Theapparat Y., Sunpaweravong S., Faroongsarng D., Otto M. (2023). Probiotic for pathogen-specific *Staphylococcus aureus* decolonisation in Thailand: A phase 2, double-blind, randomised, placebo-controlled trial. Lancet Microbe.

[33] Du X., Larsen J., Li M., Walter A., Slavetinsky C., Both A., Sanchez Carballo P.M., Stegger M., Lehmann E., Liu Y. (2021). Staphylococcus epidermidis clones express Staphylococcus aureus-type wall teichoic acid to shift from a commensal to pathogen lifestyle. Nat. Microbiol..

[34] Lee K.H., Gordon A., Shedden K., Kuan G., Ng S., Balmaseda A., Foxman B. (2019). The respiratory microbiome and susceptibility to influenza virus infection. PLoS ONE.

[35] Yildiz S., Pereira Bonifacio Lopes J.P., Bergé M., González-Ruiz V., Baud D., Kloehn J., Boal-Carvalho I., Schaeren O.P., Schotsaert M., Hathaway L.J. (2020). Respiratory tissue-associated commensal bacteria offer therapeutic potential against pneumococcal colonization. eLife.

[36] Fu J., Liu X., Cui Z., Zheng Y., Jiang H., Zhang Y., Li Z., Liang Y., Zhu S., Chu P.K. (2023). Probiotic-based nanoparticles for targeted microbiota modulation and immune restoration in bacterial pneumonia. Natl. Sci. Rev..

[37] Netea M.G., Domínguez-Andrés J., Barreiro L.B., Chavakis T., Divangahi M., Fuchs E., Joosten L.A.B., van der Meer J.W.M., Mhlanga M.M., Mulder W.J.M. (2020). Defining trained immunity and its role in health and disease. Nat. Rev. Immunol..

[38] Gaush C.R., Smith T.F. (1968). Replication and plaque assay of influenza virus in an established line of canine kidney cells. Appl. Microbiol..

