# Supplementary material for: Colonizing Bacteria Aggravate Inflammation, Cytotoxicity and Immune Defense During Influenza A Virus Infection
Source: Int J Mol Sci. 2025 Jun 3;26(11):5364. doi: 10.3390/ijms26115364 (PMC12154513; doi:10.3390/ijms26115364)
Supplement: Supplementary file 1 [file ijms-26-05364-s001.zip › ijms-3644799-supplementary.pdf]

Colonizing bacteria aggravate inflammation, cytotoxicity and immune defense during influenza A virus infection

Appendix

Supplementary Figures

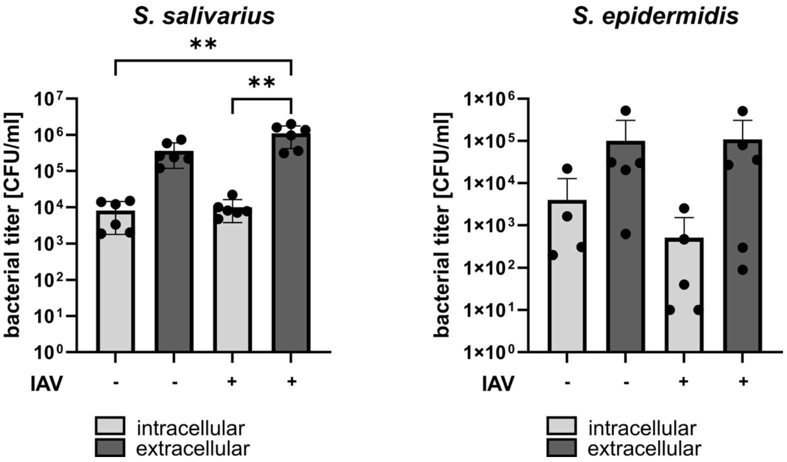

Supplementary Figure 1. Bacterial titers after colonization of the lung cell culture model colonized with commensals. Intra- and extracellular bacterial titers [colony forming units (CFU) / ml] were determined 32 h p. io. by serial dilution on Müller-Hinton-agar plates. Shown are the means + SD of at least four independent experiments with two technical replicates. Significance was determined by Kruskal-Wallis and Dunn's multiple comparison test. **p < 0.01.

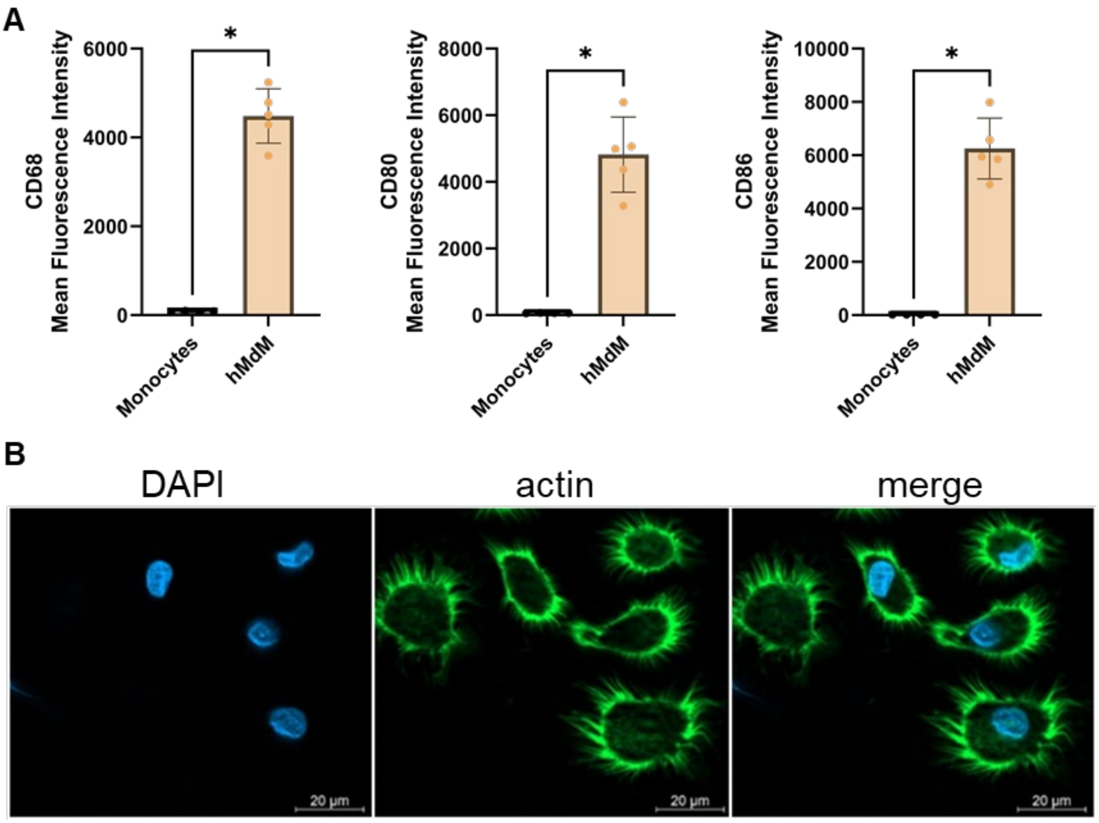

Supplementary Figure 2. Generation of hMdM. (A) For infection experiments with macrophages, primary human monocyte-derived macrophages (hMdM) were generated and the differentiation was confirmed by flow cytometric analysis of the markers CD68, CD80, and CD86. (B) Immunofluorescence revealed typical macrophage morphology. Statistical significance was determined using the Mann-Whitney test (* $p \leq 0.05$).
